# The prognostic value of routine preoperative blood parameters in muscle-invasive bladder cancer

**DOI:** 10.1186/s12894-020-00602-9

**Published:** 2020-03-19

**Authors:** Jingqi Zhang, Xiaozhou Zhou, Hua Ding, Liwei Wang, Sha Liu, Yuting Liu, Zhiwen Chen

**Affiliations:** 1 Institute of Urology, Department of Urology, First Affiliated Hospital, Army Medical University (Third Military Medical University), Chongqing, 400038 China; 2 Department of Cell Biology, College of Basic Medical Sciences, Army Medical University (Third Military Medical University), Chongqing, 400038 China

**Keywords:** Invasive-muscle bladder cancer, Neutrophil-lymphocyte ratio, Platelet-lymphocyte ratio, Hemoglobin, Prognosis

## Abstract

**Background:**

A routine blood examination is one of the most rapid, convenient and inexpensive clinical examinations that can reflect a patient’s inflammatory status and other blood conditions, and the prognostic value of routine preoperative blood parameters in MIBC patients is still unclear, so we evaluated the prognostic value of routine preoperative blood parameters in muscle-invasive bladder cancer (MIBC) following radical cystectomy (RC).

**Methods:**

Data on 202 patients with MIBC who underwent RC at our institution were retrospectively collected between October 2007 and August 2018. The median preoperative neutrophil-lymphocyte ratio (NLR), platelet-lymphocyte ratio (PLR) and hemoglobin (HGB) values were used as cutoffs to form the low and high NLR, low and high PLR, and low and high HGB groups, respectively. The clinicopathologic characteristics of each group were compared by chi-square and t tests. Kaplan-Meier survival and multivariate Cox regression analyses were used to analyze prognosis.

**Results:**

The median NLR, PLR and HGB values were 2.42, 112 and 125 g/L, respectively. Kaplan-Meier results showed that the low HGB group had poor progression-free survival (PFS), cancer-specific survival (CSS) and overall survival (OS). A high NLR and high PLR groups correlated only with poor OS. Multivariate Cox analyses showed that pathological T3/4 stage, positive lymph node status and low HGB were independent risk factors for PFS, CSS and OS, and age was the only independent risk factor for OS.

**Conclusion:**

Preoperative peripheral blood HGB is an independent risk factor for the prognosis of MIBC patients. These data suggest that HGB may be a useful prognostic marker for MIBC patients undergoing RC.

## Background

Bladder cancer is the ninth most common malignant tumor in the world and the most common malignant tumor of the urinary system [1], of which muscle-invasive bladder cancer (MIBC) accounts for approximately 25–30%. Currently, radical cystectomy (RC) is the standard treatment for MIBC, with a 5-year survival rate of 50–66% [2–4]. In recent years, there has been an urgent need to find sensitive and specific molecular markers for risk stratification in cancer patients due to the goal of individualized precision therapy. In the past, most biomarkers were determined based on PCR or immunohistochemistry, while a routine blood examination is one of the most rapid, convenient and inexpensive clinical examinations that can reflect a patient’s inflammatory status and other blood conditions through neutrophils, lymphocytes, platelets, hemoglobin and other indicators. Studies have shown that systemic inflammatory reactions associated with tumors can affect tumor progression, metastasis and prognosis [5, 6]. A number of inflammatory indicators, such as the neutrophil-lymphocyte ratio (NLR) and the platelet-lymphocyte ratio (PLR), have been paid attention to in breast cancer, colorectal cancer and gastric cancer [7–9]. In addition, studies have shown that colorectal cancer patients have poor prognoses with low preoperative hemoglobin (HGB) levels [10]. At present, the prognostic value of routine preoperative blood parameters in MIBC patients is still unclear. Therefore, our study aimed to evaluate the prognostic value of routine preoperative blood parameters in MIBC patients by analyzing the relationship between routine preoperative blood parameters and the clinicopathologic features and prognosis of MIBC patients following RC.

## Methods

### Clinical data

Clinical data and postoperative follow-up results of bladder cancer patients admitted to the Urology Department of the First Affiliated Hospital of Army Medical University were retrospectively collected between October 2007 and August 2018. The inclusion criteria were as follows: **①** the patient underwent RC; **②** the patient was pathologically diagnosed with muscle-invasive urothelial carcinoma of the bladder; **③** the preoperative imaging examination excluded distant metastasis; and **④** perfect patient information. The exclusion criteria were as follows: **①** the patient suffered from other malignant tumors; **②** the presence of acute and chronic inflammation or diseases of the blood or immune system before the preoperative blood draw; **③** the patient underwent neoadjuvant chemotherapy or adjuvant chemotherapy; and **④** follow-up time was less than 3 months. A total of 202 patients were enrolled, including 140 patients in stage T2, 33 patients in stage T3, and 29 patients in stage T4.

### The research group

The NLR and PLR were calculated by determining the ratio of the neutrophil absolute value to the lymphocyte absolute value and the platelet absolute value to the lymphocyte absolute value, respectively. The median NLR, PLR and HGB values were 2.42, 112 and 125 g/L, respectively, which were chosen as cutoffs to form the low and high NLR, low and high PLR, and low and high HGB groups, respectively. The relationship between the three groups and the clinicopathologic features and prognosis of MIBC patients was further analyzed.

### Clinical variables and follow-up results

General information and the pathological stage, pathological grade, lymph node status, preoperative neutrophil value, preoperative lymphocyte value, preoperative platelet value and preoperative hemoglobin value of MIBC patients were recorded in detail. The pathological stage of bladder cancer followed the tumor node metastasis (TNM) staging of the American Joint Committee on Cancer (AJCC), and pathological grade was adopted by the World Health Organization (WHO) in 2004. The main outcome index of this study was CSS, and the secondary indexes were PFS and OS. The follow-up deadline was December 1, 2018.

### Statistical analysis

IBM SPSS 20.0 software was used for statistical analyses. Continuous variables were presented as the mean ± standard deviation, and were compared by the t test. Categorical variables were expressed as cases and percentages, and were compared by the *χ*^2^ test. Kaplan-Meier survival and multivariate Cox regression analyses were used to analyze prognosis. A *P* value < 0.05 was considered statistically significant.

## Results

### Patient characteristics

The follow-up time of the patients ranged from 3 to 138 months, and the median follow-up time was 30 months. The age was 64.98 ± 9.73 years, and the male to female ratio was 4.9:1. All patients had negative surgical margins.

### Results of single factor analysis

A t test was conducted to examine the continuous variables of the low and high NLR, low and high PLR, and low and high HGB groups, and *χ*^2^ test was conducted to examine the categorical variables. There were statistically significant differences in gender, pathological grade, pathological T stage and positive lymph node status between the low and high NLR groups. There were statistically significant differences in pathological T stage between the low and high PLR groups. There were statistically significant differences in gender, pathological T stage and positive lymph node status between the low and high HGB groups. Specific single factor analysis results are shown in Table 1.

**Table 1:** Clinicalpathologic characteristics of cohort

Factors	NLR			PLR			HGB		
	NLR ≥ 2.42 (*n* = 103)	NLR < 2.42 (*n* = 99)	*P*	PLR ≥ 112 (*n* = 102)	PLR < 112 (*n* = 100)	*P*	HGB ≥ 125 (*n* = 108)	HGB < 125 (*n* = 94)	*P*
Age(x ± s) /years	66.1 ± 9.2	63.8 ± 10.2	0.094	65.4 ± 9.5	64.6 ± 10.0	0.574	63.7 ± 9.7	66.4 ± 9.7	0.050
BMI(x ± s) /kg.m^−2^	22.7 ± 3.4	23.1 ± 2.9	0.356	22.7 ± 3.5	23.1 ± 2.8	0.290	23.3 ± 3.1	22.5 ± 3.2	0.082
Gender [n(%)]			0.002*			0.660			0.001*
Male	94 (91.3)	74 (74.7)		86 (84.3)	82 (82.0)		99 (91.7)	69 (73.4)	
Female	9 (8.7)	25 (25.3)		16 (15.7)	18 (18.0)		9 (8.3)	25 (26.6)	
Smoking [n(%)]			0.411			0.329			0.535
yes	59 (57.3)	51 (51.5)		59 (57.8)	51 (51.0)		61 (56.5)	49 (52.1)	
no	44 (42.7)	48 (48.5)		43 (42.2)	49 (49.0)		47 (43.5)	45 (47.9)	
Pathological grade [n(%)]			0.006*			0.195			0.747
Low grade	13 (12.6)	28 (28.3)		17 (16.7)	24 (24.0)		21 (19.4)	20 (21.3)	
High grade	90 (87.4)	71 (71.7)		85 (83.3)	76 (76.0)		87 (80.6)	74 (78.7)	
Pathological T stage [n(%)]			0.006*			0.012*			0.044*
T2	61 (59.2)	79 (79.8)		61 (59.8)	79 (79.0)		83 (76.9)	57 (60.6)	
T3	22 (21.4)	11 (11.1)		21 (20.6)	12 (12.0)		13 (12.0)	20 (21.3)	
T4	20 (19.4)	9 (9.1)		20 (19.6)	9 (9.0)		12 (11.1)	17 (18.1)	
Lymph node [n(%)]			0.022*			0.216			0.012*
Positive	24 (23.3)	11 (11.1)		21 (20.6)	14 (14.0)		12 (11.1)	23 (24.5)	
Negative	79 (76.7)	88 (88.9)		81 (79.4)	86 (86.0)		96 (88.9)	71 (75.5)	

*NLR* Neutrophil-lymphocyte ratio, *PLR* Platelet-lymphocyte ratio, *HGB* Hemoglobin, *BMI* Body mass index; * *P* < 0.05

### Survival analysis

**Fig. 1:**
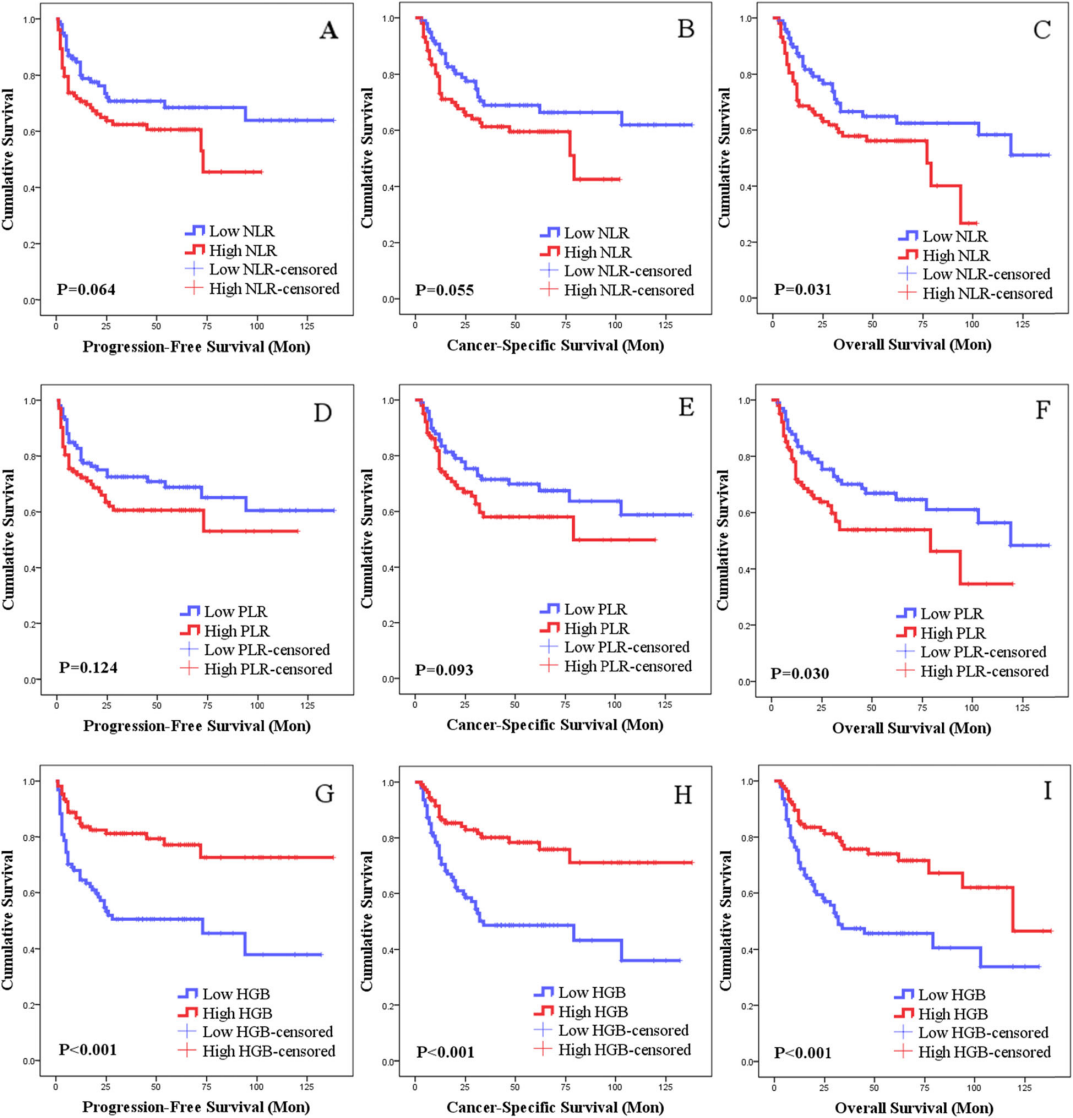
Kaplan-Meier results of PFS, CSS and OS grouped by NLR, PLR and HGB. **a**, **b**, **c**: NLR’s relationship with PFS, CSS and OS, respectively; **d**, **e**, **f**: PLR’s relationship with PFS, CSS and OS, respectively; **g**, **h**, **i**: HGB’s relationship with PFS, CSS and OS, respectively

Kaplan-Meier analysis was performed to examine the PFS, CSS and OS of the three groups, namely, the low and high NLR, low and high PLR, and low and high HGB groups, and the log-rank test was performed on the survival curves. Comparison of survival rate of NLR, PLR and HGB grouping are shown in Table 2. The log-rank test results showed that the *P* values of the PFS, CSS and OS curves of the low and high NLR groups were 0.064, 0.055 and 0.031, respectively, and the P values of the low and high PLR groups were 0.124, 0.093 and 0.030, respectively. The P values of the low and high HGB groups were all 0.000. Specific survival curve analysis results are shown in Fig. 1.

**Table 2:** Survival rate of NLR, PLR and HGB grouping

Grouping	Years	Progression-free survival rate	Cancer-specific survival rate	Overall survival rate
NLR < 2.42	1	80.0%	88.4%	87.3%
	2	73.5%	78.8%	77.9%
	5	68.5%	68.9%	64.9%
NLR ≥ 2.42	1	70.6%	73.2%	70.6%
	2	64.9%	66.5%	64.2%
	5	60.6%	59.5%	56.1%
PLR < 112	1	78.5%	85.6%	85.6%
	2	75.0%	77.8%	77.8%
	5	68.8%	69.8%	66.9%
PLR ≥ 112	1	72.2%	75.3%	71.8%
	2	63.5%	66.9%	63.8%
	5	60.6%	58.0%	53.9%
HGB < 125	1	64.5%	72.7%	70.9%
	2	54.6%	59.7%	58.2%
	5	50.6%	48.6%	45.6%
HGB ≥ 125	1	84.7%	87.4%	85.6%
	2	82.5%	84.1%	82.3%
	5	77.2%	78.3%	74.0%

*NLR* Neutrophil-lymphocyte ratio, *PLR* Platelet-lymphocyte ratio, *HGB* Hemoglobin

### Univariate and multivariate cox regression results

The univariate Cox analysis showed that pathological T3/4 stage, positive lymph node status and low HGB were risk factors influencing the PFS, CSS and OS of MIBC patients, while age, high NLR and high PLR were the only risk factors for OS. Univariate Cox regression results are shown in Table 3. These significantly different variables and clinically significant pathological grade were included in the multivariate Cox regression analysis, and the results showed that the independent risk factors influencing PFS, CSS and OS were pathological T3/4 stage, positive lymph node status and low HGB. Age was identified as the only independent risk factor affecting OS, not for PFS and CSS. Multivariate Cox regression results are shown in Table 4.

**Table 3:** Univariate Cox analysis of PFS, CSS and OS of MIBC patients

Variate	PFS		CSS		OS	
	HR(95%CI)	*P*	HR(95%CI)	*P*	HR(95%CI)	*P*
Age	1.016 (0.990–1.042)	0.238	1.017 (0.991–1.044)	0.193	1.030 (1.005–1.056)	0.021*
BMI	0.979 (0.906–1.057)	0.581	0.974 (0.902–1.052)	0.499	0.994 (0.926–1.067)	0.864
Gender(male)	1.027 (0.550–1.918)	0.933	1.030 (0.552–1.923)	0.926	1.102 (0.606–2.002)	0.751
Smoking(yes)	0.892 (0.554–1.435)	0.637	0.953 (0.592–1.535)	0.844	0.929 (0.594–1.453)	0.746
Pathological grade (high grade)	1.169 (0.648–2.111)	0.604	1.255 (0.694–2.272)	0.452	1.409 (0.794–2.498)	0.241
Pathological T stage						
T2	reference		reference		reference	
T3	3.185 (1.772–5.724)	< 0.001*	3.242 (1.802–5.833)	< 0.001*	2.926 (1.670–5.129)	< 0.001*
T4	5.824 (3.266–10.385)	< 0.001*	5.841 (3.268–10.440)	< 0.001*	5.653 (3.265–9.786)	< 0.001*
Lymph node (positive)	4.559 (2.769–7.507)	< 0.001*	4.625 (2.799–7.643)	< 0.001*	4.183 (2.581–6.780)	< 0.001*
HGB ≥ 125	0.351 (0.211–0.583)	< 0.001*	0.355 (0.214–0.591)	< 0.001*	0.422 (0.265–0.671)	< 0.001*
NLR ≥ 2.42	1.569 (0.965–2.551)	0.069	1.603 (0.982–2.617)	0.059	1.653 (1.038–2.632)	0.034*
PLR ≥ 112	1.449 (0.896–2.345)	0.131	1.504 (0.928–2.436)	0.097	1.645 (1.041–2.600)	0.033*

*NLR* Neutrophil-lymphocyte ratio, *PLR* Platelet-lymphocyte ratio, *HGB* Hemoglobin, *BMI* Body mass index, *PFS* Progression-free survival, *CSS* Cancer-specific survival, *OS* Overall survival, *HR* Hazard ratio, *CI* Confidence interval; * *P* < 0.05

**Table 4:** Mutivariate Cox analysis of PFS, CSS and OS of MIBC patients

Variate	PFS		CSS		OS	
	HR(95%CI)	*P*	HR(95%CI)	*P*	HR(95%CI)	*P*
Age	–	–	–	–	1.032 (1.006–1.059)	0.016*
Pathological T stage						
T2	reference		reference		reference	
T3	2.154 (1.109–4.187)	0.024*	2.209 (1.136–4.297)	0.020*	1.885 (1.005–3.537)	0.048*
T4	6.037 (3.245–11.233)	< 0.001*	5.604 (3.015–10.417)	< 0.001*	5.502 (3.049–9.928)	< 0.001*
Pathological grade (high grade)	0.550 (0.279–1.084)	0.084	0.610 (0.309–1.205)	0.155	0.795 (0.419–1.509)	0.483
Lymph node (positive)	3.815 (2.162–6.732)	< 0.001*	3.428 (1.926–6.099)	< 0.001*	3.187 (1.839–5.523)	< 0.001*
HGB ≥ 125	0.438 (0.261–0.735)	0.002*	0.474 (0.280–0.802)	0.005*	0.565 (0.348–0.918)	0.021*
NLR ≥ 2.42	–	–	–	–	0.969 (0.542–1.734)	0.916
PLR ≥ 112	–	–	–	–	1.101 (0.631–1.921)	0.734

*NLR* Neutrophil-lymphocyte ratio, *PLR* Platelet-lymphocyte ratio, *HGB* Hemoglobin, *BMI* Body mass index, *PFS* Progression-free survival, *CSS* Cancer-specific survival, *OS* Overall survival, *HR* Hazard ratio, *CI* Confidence interval; * *P* < 0.05

## Discussion

A routine blood examination is one of the most commonly used tests in clinical practice. It mainly measures the white blood cell system, the platelet system and the hemoglobin system and can quickly provide information about the states of inflammation and anemia. Evidence suggests that peripheral blood analysis can reflect the microenvironmental status of tumors [11, 12]. For example, the NLR, PLR and other inflammatory indicators can be used to predict the prognosis of tumors. In addition, tumor-related anemia has gradually been paid attention to because the hypoxic environment of the body is likely to promote tumor growth and invasion [13]. Currently, the prognostic value of these routine blood indexes in MIBC patients is still unclear. Therefore, this study further explored the value of routine preoperative blood indexes in MIBC patients by analyzing the relationship between the preoperative NLR, PLR and HGB and clinicopathologic characteristics and prognosis.

Studies have shown that inflammation affects the occurrence, development and metastasis of tumors [11], and cancer-related inflammation is a key determinant of prognosis in cancer patients, while tumor patients with systemic inflammation have a poor prognosis [6]. The systemic inflammatory response can affect the nutritional status, immune function and psychological fluctuations of patients, and an imbalance in the above factors will affect prognosis. In recent years, a number of studies have been looking for factors that can truly reflect the systemic inflammatory response in patients and exploring their value in risk stratification in tumor patients, such as C-reactive protein, the NLR, the PLR and the scoring system (GPS score) formed by combining multiple inflammatory factors [14, 15]. Studies showed that the NLR was increased in patients undergoing RC with advanced or invasive diseases, manifested by increased tumor staging and lymph node metastasis [16, 17]. In addition, preoperative increases in the NLR in patients with RC were associated with a poor prognosis [18, 19]. However, a high NLR and a high PLR of this study were not identified as independent risk factors affecting PFS, CSS and OS in the multivariate Cox analysis, speculating that it may be removed by the interference of other factors in the multivariate Cox regression analysis. Therefore, the value of the NLR and PLR needs to be evaluated by multicenter and large sample studies. Furthermore, Leibowitz-Amit [20] found that MIBC patients with a high lymphocyte absolute value before neoadjuvant chemotherapy (NAC) were associated with a high complete response rate, while the complete response rate in patients with a high NLR and PLR was low. Black et al. [21] also found that high NLR was associated with a decreased response to NAC and shorter prognosis in MIBC patients, which suggests that the inflammatory response affects the body’s sensitivity to chemotherapy and the anti-inflammatory treatment before chemotherapy may play an auxiliary role.

The prognosis of cancer patients is dependent not only on the biological characteristics of tumors, to a greater extent, but also on host reactions [5, 6]. Motomura et al. [22] found that a high NLR was associated with increased serum and peritumor IL-17 and VEGF levels, suggesting that the systemic inflammatory response may further affect tumor development through the upregulation of proinflammatory cytokines. Grivennikov et al. [11] concluded that immune cells can affect tumor cells by producing cytokines, chemokines, growth factors, prostaglandins, reactive oxygen species, etc. Studies have also examined the relationship between macrophages and tumor cells. Chen et al. [23] found that PTEN mutation or deletion can promote the infiltration of macrophages in glioblastoma, while macrophages migrating to the tumor can secrete the SPP1 factor to promote the survival of tumor cells and the formation of new blood vessels, thereby promoting tumor progression. In conclusion, further studies on the regulation of systemic inflammatory responses by the tumor and host-derived factors may provide new therapeutic strategies for cancer patients.

A previous epidemiological investigation found that 67 to 80% of patients with bladder cancer presented with a painless gross hematuria [24], which was one of the main reasons for the decrease in HGB before surgery. In addition, bone marrow suppression caused by neoadjuvant chemotherapy and tumor invasion also led to a decrease in HGB. A meta-analysis showed that low preoperative HGB levels were considered prognostic factors for MIBC patients with RC [25]. Grimm et al. [26] found that low preoperative HGB levels were independent risk factors for CSS and OS after RC. Bi et al. [27] found that patients with high preoperative HGB levels and a high body mass index (BMI) had a good postoperative prognosis after RC, and one study showed that HGB was positively correlated with BMI [28]. Preoperative low hemoglobin levels can lead to tumor hypoxia, which promotes tumor growth by stimulating angiogenesis, acquiring genome mutations, and increasing resistance to apoptosis [13, 29] and further leads to increased staging and a poor prognosis. On the other hand, it is speculated that tumor-related inflammation may result in the release of various inflammatory factors, which may affect erythropoietin synthesis and lead to the decrease in HGB [30]. Currently, hemoglobin is not commonly used in various risk prediction tools, so the results of this study suggest that we need to improve our understanding of the impact of preoperative anemia or low hemoglobin levels on postoperative survival.

There are still some limitations to this study. (1) This study was a retrospective study, and patient selection may cause bias; (2) Some patients were followed up by telephone with the possibility of inaccurate outcomes; (3) Only the NLR, PLR and HGB were examined in this study, while other blood indicators may be of greater value in the prognosis of bladder cancer.

## Conclusion

The preoperative peripheral blood HGB level is an independent risk factor for the prognosis of MIBC patients, and it can be used as a prognostic indicator for MIBC patients following RC.

## Data Availability

The datasets analyzed during the current study is available from the corresponding author on reasonable request.

## Abbreviations

NLR: Neutrophil-lymphocyte ratio
PLR: Platelet-lymphocyte ratio
HGB: Hemoglobin
BMI: Body mass index
PFS: Progression-free survival
CSS: Cancer-specific survival
OS: Overall survival
HR: Hazard ratio
CI: Confidence interval
MIBC: Muscle-invasive bladder cancer
RC: Radical cystectomy
